# The Relationship between Resistance Exercise Performance and Ventilatory Efficiency after Beetroot Juice Intake in Well-Trained Athletes

**DOI:** 10.3390/nu13041094

**Published:** 2021-03-27

**Authors:** Noemí Serra-Payá, Manuel Vicente Garnacho-Castaño, Sergio Sánchez-Nuño, Lluís Albesa-Albiol, Montserrat Girabent-Farrés, Luciana Moizé Arcone, Alba Pardo Fernández, Adrián García-Fresneda, Jorge Castizo-Olier, Xavier Viñals, Lorena Molina-Raya, Manuel Gomis Bataller

**Affiliations:** 1 School of Health Sciences, TecnoCampus Pompeu Fabra University, Ernest Lluch, 32 (Porta Laietana), 08302 Mataró-Barcelona, Spain; nserra@tecnocampus.cat (N.S.-P.); ssanchezn@tecnocampus.cat (S.S.-N.); lalbesa@tecnocampus.cat (L.A.-A.); mgirabent@tecnocampus.cat (M.G.-F.); lmoize@tecnocampus.cat (L.M.A.); apardo@tecnocampus.cat (A.P.F.); agarciaf@tecnocampus.cat (A.G.-F.); jcastizo@tecnocampus.cat (J.C.-O.); xvinals@tecnocampus.cat (X.V.); mgomis@tecnocampus.cat (M.G.B.); 2 Campus Docent Sant Joan de Déu, Fundación Privada, 08304 Barcelona, Spain; lmolina@santjoandedeu.edu.es

**Keywords:** VE•VCO_2_^−1^ slope, oxygen uptake efficiency slope, partial pressure of end-tidal carbon dioxide, nitric oxide, nitrate, nitrite

## Abstract

The assessment of ventilatory efficiency is critical to understanding the matching of ventilation (VE) and perfusion in the lungs during exercise. This study aimed to establish a causal physiological relationship between ventilatory efficiency and resistance exercise performance after beetroot juice (BJ) intake. Eleven well-trained males performed a resistance exercise test after drinking 140 mL of BJ (~12.8 mmol NO_3_^−^) or a placebo (PL). Ventilatory efficiency was assessed by the VE•VCO_2_^−1^ slope, the oxygen uptake efficiency slope and the partial pressure of end-tidal carbon dioxide (PetCO_2_). The two experimental conditions were controlled using a randomized, double-blind crossover design. The resistance exercise test involved repeating the same routine twice, which consisted of wall ball shots plus a full squat (FS) with a 3 min rest or without a rest between the two exercises. A higher weight lifted was detected in the FS exercise after BJ intake compared with the PL during the first routine (*p* = 0.004). BJ improved the VE•VCO_2_^−1^ slope and the PetCO_2_ during the FS exercise in the first routine and at rest (*p* < 0.05). BJ intake improved the VE•VCO_2_^−1^ slope and the PetCO_2_ coinciding with the resistance exercise performance. The ergogenic effect of BJ could be induced under aerobic conditions at rest.

## 1. Introduction

The assessment of ventilatory efficiency is critical to understanding the matching of ventilation (VE) and perfusion in the lungs. Increased VE/perfusion mismatching induces a steepened inefficiency of the pulmonary gas exchange. In this regard, an increase in VE occurs for a given production of carbon dioxide (VCO_2_) and arterial partial pressure of CO_2_ (PCO_2_) essentially contributing to hyperpnea and dyspnea [1].

During high intensity exercise, the enhancement of VE and the removal VCO_2_ are crucial for the homeostatic control of the pH [2] and arterial hypoxemia [3] induced by lactic acidosis [4]. It has been widely reported that high intensity exercise reduces the efficiency of the pulmonary gas exchange [5,6] resulting in an augmented VE/perfusion mismatching [7]. The aggravated VE/perfusion during exercise could affect the perfusion of the limb locomotor muscle, reducing exercise tolerance [2].

Ventilatory efficiency is commonly assessed by determining the slope of the linear relationship between VE and VCO_2_ (VE•VCO_2_^−1^ slope) [8,9] and the oxygen uptake efficiency slope (OUES) [10]. The OUES is defined as the rate of the increase of oxygen uptake (VO_2_) in response to VE during exercise and reflects the efficiency of the extraction and introduction of oxygen into the body [10]. The VE•VCO_2_^−1^ slope is considered to be a gold standard method in pathological events [11] and the OUES is actually used to predict mortality and ventilatory inefficiency in cardiorespiratory disease [12,13,14,15,16] and to determine exercise tolerance [12,17]. In addition, the partial pressure of end-tidal carbon dioxide (PetCO_2_) is a noninvasive and suitable marker for assessing the VE/perfusion relationship and ventilatory efficiency particularly in cardiac and lung diseases [18,19,20].

The VE•VCO_2_^−1^ slope and the OUES have been used to a lesser extent for assessing ventilatory efficiency in physically active and healthy people [8] and in highly trained athletes in more endurance-type exercises [2]. The role of the VE•VCO_2_^−1^ slope and the OUES for exercise performance is unclear [2,21]. In addition, ventilatory efficiency is conditioned by the type of exercise test [8], gender and the fitness of the participants [22].

The assessment of ventilatory efficiency is not a common procedure during resistance exercise. Recently, our research group demonstrated that ventilatory efficiency, assessed by the VE•VCO_2_^−1^ slope and the OUES, was similar between a cycle ergometer and a half-squat exercise at the same metabolic intensity in a predominantly aerobic pathway [23]. The VE•VCO_2_^−1^ slope and the OUES during high intensity resistance exercises in a mainly anaerobic metabolism remain unknown. It is plausible to propose a marked ventilatory inefficiency under an environment of low oxygen availability and high blood lactate concentrations during high intensity resistance exercise as occurs in endurance exercise [7]. However, no attention has been paid to the assessment of the VE•VCO_2_^−1^ slope and the OUES in well-trained resistance training practitioners during high intensity resistance exercise.

Nitric oxide (NO) is a gaseous signaling molecule that plays a key role in lung physiology [24]. Exogenous NO precursors (sildenafil, L-arginine, inhaled NO, beetroot juice (BJ)) have been used for improving ventilatory efficiency in cardiovascular and respiratory diseases [25,26,27,28]. The mechanisms of clinical improvement were related, at least in part, to improvements in pulmonary vasodilation [29,30], pulmonary blood flow and more uniform VE/perfusion matching [20]. It not usual to assess the effects of exogenous NO precursors on ventilatory efficiency (the VE•VCO_2_^−1^ slope and the OUES) in well-trained athletes.

Concretely, BJ has been demonstrated to improve exercise efficiency [31], lung function and performance during cardiopulmonary exercise testing (CPET) in more endurance-type exercises through the nitrate (NO_3_^−^) to nitrite (NO_2_^−^) conversion pathway [32]. In many cases, improvements in the lung function after BJ intake are often not associated with improvements in endurance performance essentially in well-trained athletes [33]. Recently, we demonstrated that BJ increased anaerobic performance only after the recovery time between exercises during a high intensity resistance exercise test in well-trained athletes. We also observed that BJ did not improve performance compared with the placebo group when anaerobic conditions were increased with no rest between resistance exercises [34]. The effects of BJ on the performance of well-trained athletes led to discrepancies among researchers. If exogenous NO precursors improve pulmonary blood flow and more uniform VE/perfusion matches [20] and high intensity exercise contributes to impaired ventilatory efficiency [7], it is likely that drinks such as BJ could induce an enhancement in performance by improving ventilatory efficiency when a rest is established between resistance exercises. To the best of our knowledge, no studies have assessed the relationship between ventilatory efficiency (VE•VCO_2_^−1^ slope and OUES) and high intensity resistance exercise performance in well-trained athletes after BJ intake.

This study aimed to establish a causal physiological association between ventilatory efficiency (VE•VCO_2_^−1^ slope and OUES), the PetCO_2_ and high intensity resistance exercise performance after acute BJ intake. Bearing in mind that high intensity exercise reduces the efficiency of the pulmonary gas exchange [5,6] resulting in an increased VE-perfusion mismatch [7], we hypothesize that NO via dietary BJ could contribute, at least in part, to delaying fatigue and increasing exercise tolerance by improving the VE•VCO_2_^−1^ slope, the OUES and the PetCO_2_ when a rest is established between resistance exercises.

## 2. Materials and Methods

### 2.1. Study Design

Participants were required to visit the Fitness Center and Exercise Physiology laboratory in four sessions over three weeks.

In session 1, the experimental procedures were clarified to the participants. During the second session, participants carried out a one-repetition maximum (1RM) test of a full squat (FS) exercise (see below for details). Both sessions were performed in the Fitness Center during the first week.

In sessions 3 and 4 (next two weeks), the two experimental conditions, BJ and the placebo (PL), were compared at the same time of day (±30 min) and under similar ambiental conditions (temperature ~22 °C, relative humidity ~50%). A one-week washout period was established between both sessions. For this purpose, the participants repeated the same resistance exercise test twice. BJ and the PL were administered three hours before the resistance exercise test in a randomized, double-blind, crossover design. Participants were completely familiarized with the resistance exercises.

**Figure 1:**
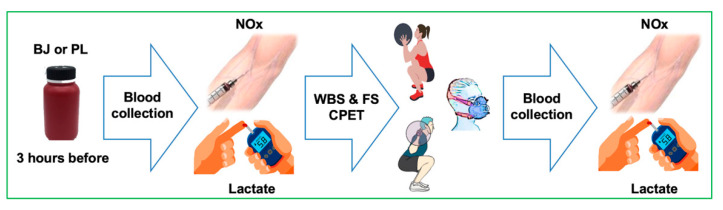
Procedures during the resistance exercise test after beetroot juice (BJ) intake and the placebo (PL) condition of the third and fourth session/BJ or PL randomized, double-blind, crossover design. Abbreviations used: BJ: beetroot juice; CPET: cardiopulmonary exercise test; FS: full squat; NOx: nitrate plus nitrite; PL: placebo; WBS: wall ball shots.

Three hours after the BJ or PL ingestion, blood was drawn to determine NO_3_^−^ plus NO_2_^−^ (NOx) concentrations and lactate at rest (pre-test) and after (post-test) the resistance exercise test. During the resistance exercise test (sessions 3 and 4), the pulmonary gas exchange to determine ventilatory efficiency (VE•VCO_2_^−1^ slope and OUES), PetCO_2_ and heart rate were monitored (Figure 1).

### 2.2. Participants

Eleven male athletes well-trained in resistance exercises were recruited for the study (age = 29.2 ± 3.7 years; height = 175.1 ± 6.3 cm; body mass = 78.9 ± 5.4 kg). The inclusion criteria included: no musculoskeletal injuries and/or diseases; more than two years of experience in resistance training; 1RM in FS equal to or greater than 120 kg; a regional, national and/or international competition level; no smoking; no consumption of any other supplement at the time of the study; no consumption of drugs or medication. The sample size was determined from the results of a pilot study involving 10 well-trained students. The calculation of the sample size was carried out as follows: α = 0.05 (5% probability of a type I error) and 1 − β = 0.80 (80% power).

Informed written consent for participation was gained from all participants. Approval was granted by the Ethics Committee of the TecnoCampus Pompeu Fabra University (IRB approval 56/2019) according to the principles and policies of the Declaration of Helsinki for research in humans.

### 2.3. Diet Control and Beetroot Juice Intake

A nutrition professional controlled the BJ and PL intake and the diet according to the guidelines established in previous studies [34,35]. The diet control was established in a food diary 48 h before sessions 3 and 4. All participants followed a similar diet 48 h before starting the resistance exercise test consisting of ~60% carbohydrates (5.5 g carbohydrate per kg), 25% lipids and 15% protein. The diet was registered by the participants 48 h before the first and the second experimental test. The same diet was replicated before both sessions (BJ and PL). Compliance with the dietary instructions was corroborated by consulting the diaries of the participants. Participants avoided foods with a high NO_3_^−^ content at least 72 h prior to the test sessions. Therefore, a food list was provided to all participants (beetroot, spinach, arugula, lettuce, celery, parsley, turnip, endives, leak, cabbage). Furthermore, the ingestion of alcohol, caffeine (except breakfast coffee) or other supplements was strictly prohibited to avoid any interaction with the BJ. Participants were requested to abstain from chewing gum, eating sweets, using a mouthwash or brushing their teeth (chlorhexidine or xylitol) 24 h before the study [36]. Participants were advised of the possible side effects of ingesting BJ such as gastrointestinal symptoms and a red appearance of feces and urine.

As in a previous study [34], BJ or the PL was ingested 3 h before the start of sessions 3 and 4. The participants were provided with a randomly assigned garnet red plastic bottle containing 140 mL of BJ Beet-It-Pro Elite Shot concentrate (~12.8 mmol, ~808 mg NO_3_^−^) (Beet IT; James White Drinks Ltd., Ipswich, UK) or the PL. The PL beverage was prepared by dissolving 2 g of powdered BJ (~0.01 mmol, 0.620 mg of NO_3_^−^, Experience-Naturgreen, Murcia, Spain) in a liter of mineral water by a nutrition professional. Lemon juice was added to simulate the flavor of the commercial drink.

### 2.4. Resistance Exercise Tests

Resistance exercise tests were carried out according to the guidelines established in a previous study [34]. A 1RM test was executed in a FS exercise to determine the loading intensity at 50% of 1RM in each participant (session 2). After a general and specific warm-up, an incremental test using increasing weights with an Olympic bar was performed to determine 1RM in a FS (in kg) exercise. The 1RM test was considered as the highest weight lifted by each participant. A 4 min rest was established between each set.

In sessions 3 and 4, resistance exercise tests were executed for the comparison of the two experimental conditions (BJ and the PL). For this purpose, two weightlifting exercises were selected: wall ball shots (WBS) and a FS. The resistance exercise test consisted of repeating the same routine twice. The first routine consisted of 90 s of WBS plus 60 s of FS with a 3 min rest between both exercises. The second routine consisted of WBS for 90 s plus FS for 60 s without a rest between the two exercises. A 3 min rest was applied between both routines. The same routine with and without a rest was selected to demonstrate the possible ergogenic effect of NOx during a rest under more aerobic conditions. We previously demonstrated that BJ increased performance to a greater extent than a placebo when a rest was established between exercises. Without a rest between exercises, the anaerobic conditions of the routine increased and there was no ergogenic effect of BJ [34].

The FS exercise was executed with free weight at a loading intensity of 50% of 1RM. The WBS were executed with a 10 kg medicine ball. The performance goal was to achieve the highest number of repetitions in each routine. The resistance exercise performance was assessed by the total weight lifted (in kg). The weight lifted was computed as the load lifted (in kg) multiplied by the number of repetitions performed during the two experimental conditions.

Participants did not perform high intensity physical exercise from 72 h before the test sessions. Participants abstained from physical exercise during the 24 h prior to beginning each test session.

### 2.5. Plasma NOx Concentrations

Blood samples were drawn from the antecubital vein in a 10 mL EDTA Vacutainer tube. Plasma was then obtained, centrifuged at 2500× *g* for 15 min, aliquoted and stored at −80 °C until the posterior analysis.

NO_3_^−^ plus NO_2_^−^ concentrations (NOx) were measured in plasma previously centrifuged at 14,000× *g* for 60 min and ultrafiltered by a 10 kDa cutoff filter (Millipore-Merck, Darmstadt, Germany). NO_3_^−^ was converted to nitrite using NO_3_^−^ reductase and the total NO_2_^−^ amount was measured by the Griess reaction using a colorimetric assay kit (Cayman Chemical Co., Ann Arbor, MI, USA). Values were expressed as NOx μM of plasma.

### 2.6. Pulmonary Gas Exchange

Pulmonary gas exchange data were recorded during the resistance exercise test (sessions 3 and 4) using a breath-by-breath open-circuit gas analyzer (Ergostik, Geratherm Respiratory, Bad Kissingen, Germany) that was calibrated before each test according to the manufacturer’s instructions. VO_2_, VE, VCO_2_, the respiratory exchange ratio (RER) and PetCO_2_ were monitored. The heart rate was checked every 5 s by telemetry (Polar Electro OY, Kempele, Finland).

Ventilatory efficiency was calculated as follows [23]:

The VE•VCO_2_^−1^ slope was established as the slope of the relationship between VE and VCO_2_.

The OUES was determined as the relationship between VO_2_ and the logarithm of the VE. VO_2_ = a log_10_VE + b.

### 2.7. Blood Lactate

Blood lactate concentrations were assessed as in previous studies [34,37,38]. The analyzer Lactate ProTM 2 (Arkray Factory Inc., KDK Corporation, Shiga, Japan) was used to determine blood lactate concentrations. Lactate ProTM 2 has shown excellent reliability and accuracy [39]. Blood samples were acquired from the index finger of the left hand.

### 2.8. Statistical Analysis

The normal distribution of the data was examined by the Shapiro–Wilk test. Data were presented as mean and standard deviation (SD) and mean and confidence intervals (95% CI). To identify significant differences between the experimental groups (BJ vs. the PL), a two-way analysis of variance (ANOVA) with repeated measures was applied (experimental condition•time). When appropriate, Bonferroni adjustments were applied for multiple comparisons. The magnitude of the response to both experimental conditions was estimated by a partial eta-squared (η_p_^2^). The scale for classification of η_p_^2^ was 0.10 = small, 0.25 = medium, 0.40 = large [40]. The statistical power (SP) was calculated. A power analysis indicated that a sample size of 10 was necessary to provide a statistical power of 0.80 or greater for the total weight lifted and ventilatory efficiency data.

Intraclass correlation coefficients (ICCs) and coefficients of variation (CVs) percentages were used to determine the relative and absolute reliability of the cardiorespiratory variables. The significance was set at *p* < 0.05. All statistical tests were performed using the software package SPSS version 25.0 for Mac (SPSS Inc., Chicago, IL, USA).

## 3. Results

The exercises and dietary practices were maintained according to the exercise and dietary guidelines. BJ and the PL were drunk at the scheduled time. Participants were blinded to the intake condition. All participants were not able to distinguish between BJ and the PL condition during the first session. Three athletes were suspicious about the intake of the PL. The dietary BJ was well-tolerated by all participants; however, a few participants had beeturia (red urine) and red stools after BJ intake. All participants did not have beeturia and red stools after the PL intake. Plasma NOx levels were increased after BJ intake (397.63 μM) compared with the PL (31.15 μM) (*p* < 0.001).

The reliability of the measurements was assessed in cardiorespiratory variables. ICCs for VO_2_, VE, VCO_2_ and PetCO_2_ were significant (*p* < 0.001). ICCs were 0.98 (CI 95%: 0.97–0.99) for VO_2_, 0.97 (CI 95%: 0.94–0.98) for VE, 0.93 (CI 95%: 0.87–0.96) for VCO_2_ and 0.97 (CI 95%: 0.95–0.98) for PetCO_2_. The CVs were 4.4% for VO_2_, 6.4% for VE, 5.3% for VCO_2_ and 3.1% for PetCO_2._

### 3.1. Resistance Exercise Performance

**Figure 2:**
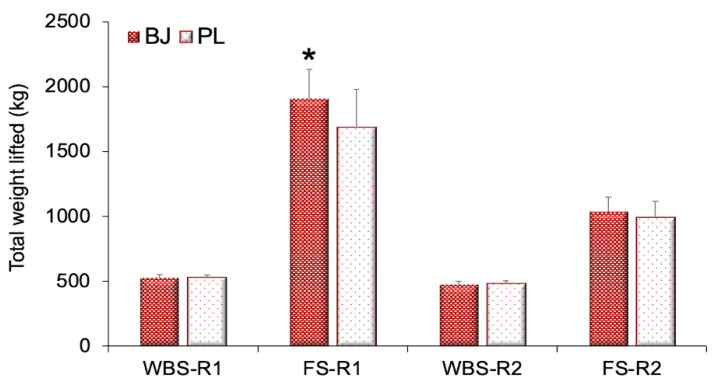
Total weight lifted (*n* = 11). Abbreviations used: BJ: beetroot juice; FS-R1: full squat during the first routine; FS-R2: full squat during the second routine; PL: placebo; WBS-R1: wall ball shots during the first routine; WBS-R2: wall ball shots during the second routine. Data are provided as mean and error bars as 95% confidence intervals. * Significant increase in the total weight lifted after BJ intake compared with the PL condition in the first routine (with a 3 min rest) (*p* = 0.004).

For the total weight lifted, a significant interaction effect was verified (F_(4,40)_ = 10.30, *p* < 0.001, η_p_^2^ = 0.51, SP = 0.99). Significant effects were found in the time (F_(4,40)_ = 119.42, *p* < 0.001, η_p_^2^ = 0.92, SP = 1.00) and the experimental condition (F_(1,10)_ = 14.00, *p* = 0.004, η_p_^2^ = 0.58, SP = 0.92). A Bonferroni test confirmed a higher weight lifted in the FS exercise after BJ intake than in the PL during the first routine (*p* = 0.004). No significant differences were found in the total weight lifted between the two experimental conditions in the second routine in the FS and WBS exercises (*p* > 0.05) (Figure 2).

### 3.2. Ventilatory Efficiency

For the VE•VCO_2_^−1^ slope, a significant interaction effect was confirmed (F_(4,40)_ = 3.48, *p* = 0.016, η_p_^2^ = 0.26, SP = 0.82). A time effect was found (F_(4,40)_ = 35.48, *p* < 0.001, η_p_^2^ = 0.78, SP = 1.00); however, no experimental condition effect was detected (*p* > 0.05).

**Figure 3:**
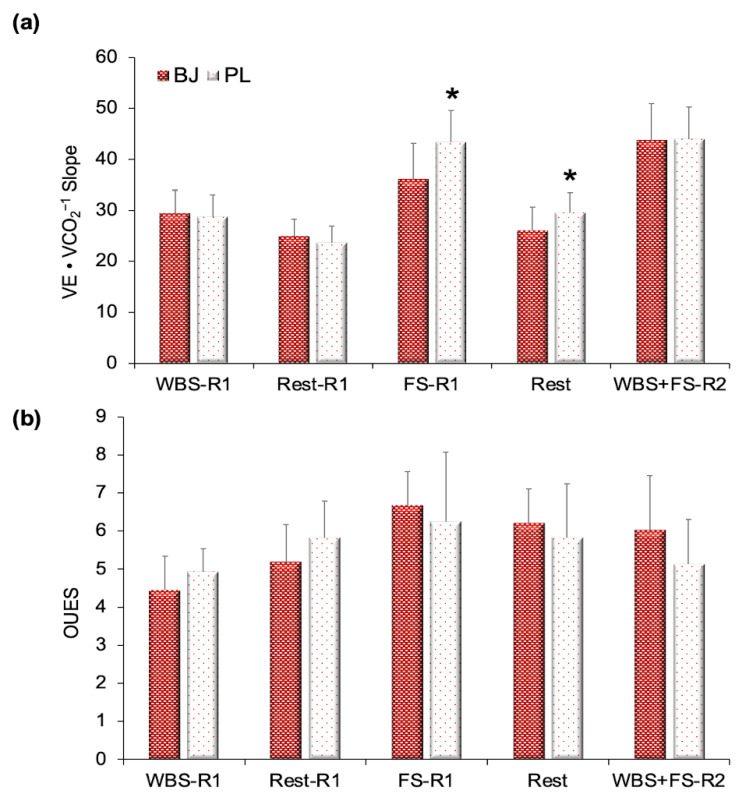
Differences between beetroot juice intake and the placebo (*n* = 11). (**a**) VE•VCO_2_^−1^ slope (**b**) OUES. Abbreviations used: BJ: beetroot juice; FS-R1: full squat during the first routine; OUES: oxygen uptake efficiency slope; PL: placebo; WBS-R1: wall ball shots during the first routine; WBS+FS-R2: wall ball shots plus full squat during the second routine (without a rest). Data are provided as mean and error bars as 95% confidence intervals. * Significant increase in the VE•VCO_2_^−1^ slope after the PL intake compared with BJ (*p* < 0.05).

A Bonferroni test verified that BJ decreased the VE•VCO_2_^−1^ slope during the FS exercise in the first routine (*p* = 0.047) and at rest between the first and the second routine (*p* = 0.009) (Figure 3a).

For the OUES, no interaction and experimental condition effects were found (*p* > 0.05) but a time effect was confirmed (F_(4,40)_ = 3.52, *p* = 0.015, η_p_^2^ = 0.26, SP = 0.82) (Figure 3b).

**Figure 4:**
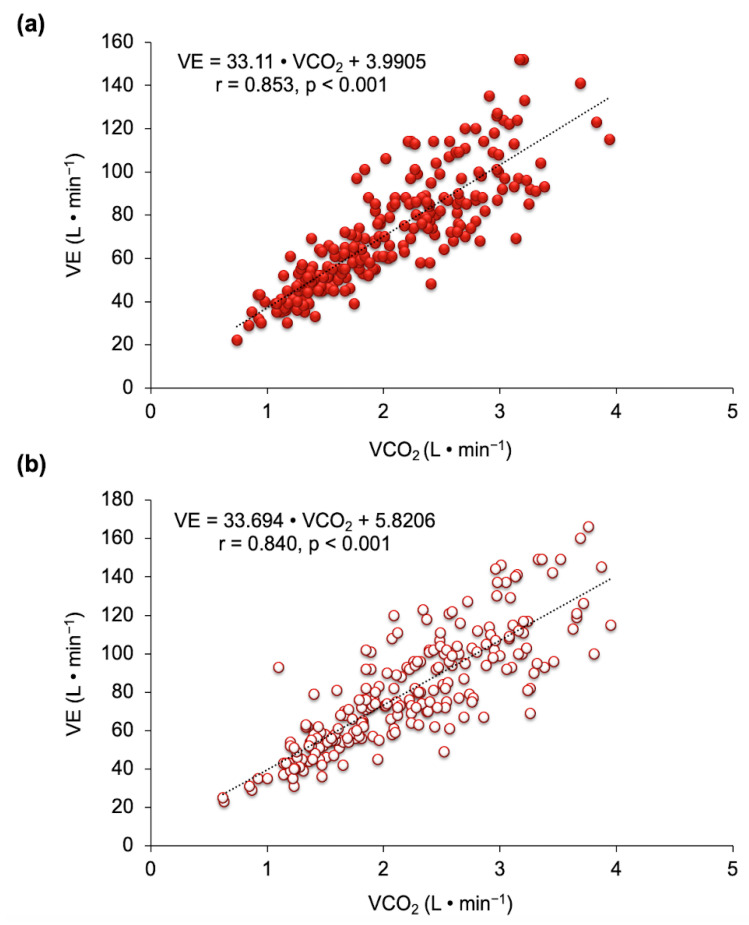
Relationship between ventilation (VE) and carbon dioxide (VE•VCO_2_^−1^ slope) after beetroot juice (**a**) and a placebo (**b**) (*n* = 11).

In the VE•VCO_2_^−1^_2_ slope, VE and VCO_2_ were highly correlated (*p* < 0.001) in the two experimental conditions (Figure 4).

**Figure 5:**
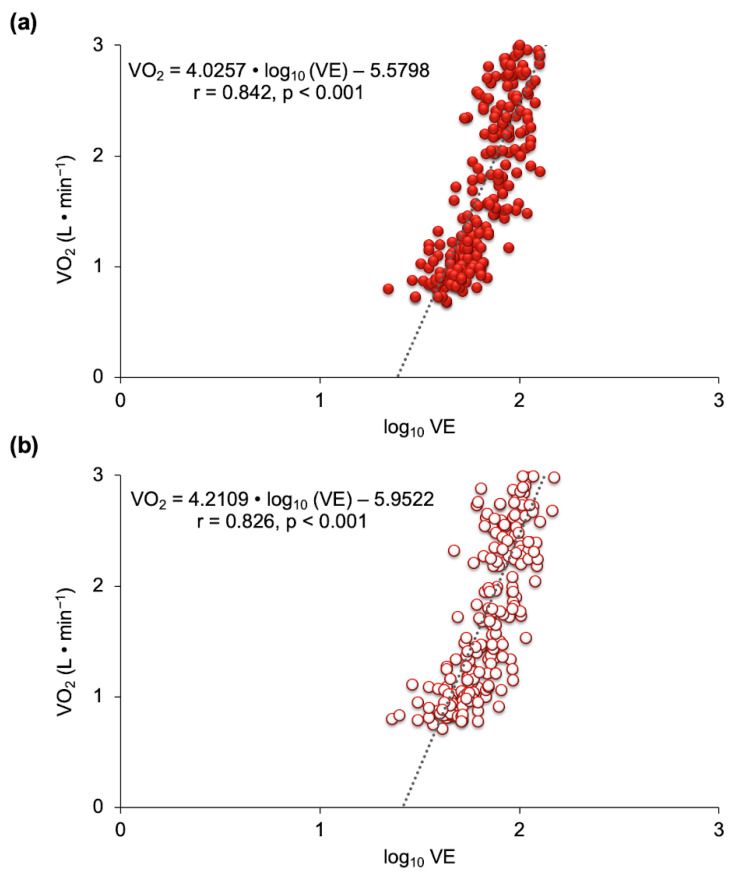
Relationship between oxygen uptake (VO_2_) log_10_ ventilation (VE) (OUES) after beetroot juice (**a**) and a placebo (**b**) (*n* = 11).

Similarly, high correlations (*p* < 0.001) were found between VO_2_ and log_10_ VE in the OUES in the two experimental conditions (Figure 5).

**Figure 6:**
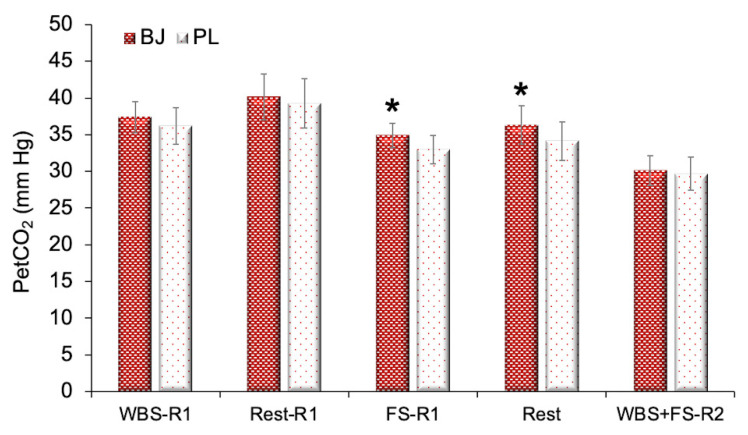
Differences between beetroot juice intake and the placebo in the partial pressure of end-tidal carbon dioxide (PetCO_2_) (*n* = 11). Abbreviations used: BJ: beetroot juice; FS-R1: full squat during the first routine; PL: placebo; WBS-R1: wall ball shots during the first routine; WBS+FS-R2: wall ball shots plus full squat during the second routine (without a rest). Data are provided as mean and error bars as 95% confidence intervals. * Significant increase in the PetCO_2_ after BJ intake compared with PL (*p* < 0.001).

For the PetCO_2_, a significant interaction effect was found (F_(4,40)_ = 3.79, *p* = 0.01, η_p_^2^ = 0.28, SP = 0.85). Time and experimental condition effects were detected (F_(4,40)_ = 58.58, *p* < 0.001, η_p_^2^ = 0.85, SP = 1.00; F_(1,10)_ = 25.01, *p* < 0.001, η_p_^2^ = 0.71, SP = 0.99, respectively). A Bonferroni adjustment corroborated that BJ increased the PetCO_2_ during the FS in the first routine (*p* < 0.001) and at rest between the first and the second routine (*p* < 0.001) (Figure 6).

### 3.3. Blood Lactate Concentrations

For the blood lactate concentrations, no interaction and experimental condition effects were discovered (*p* > 0.05); nevertheless a time effect was detected (F_(4,40)_ = 535.26, *p* < 0.001, η_p_^2^ = 0.98, SP = 1.00).

## 4. Discussion

The principal finding of this study was that BJ intake improved the total weight lifted, the VE•VCO_2_^−1^ slope and the PetCO_2_ in the FS exercise during the first routine and during the rest between both routines in well-trained athletes; conversely, increased plasma NOx concentrations had no effect on the OUES and did not improve the resistance exercise performance, the VE•VCO_2_^−1^ slope and the PetCO_2_ when anaerobic conditions were increased during the second routine. Consistent with the experimental hypothesis, we suggest a causal physiological relationship between the VE•VCO_2_^−1^ slope, the PetCO_2_ and the total weight lifted in the FS exercise after acute BJ intake when a rest between WBS and FS exercises was applied.

The assessment of the VE•VCO_2_^−1^ slope and the OUES in resistance exercises has not received the same consideration as, for instance, in endurance exercises. We previously demonstrated that resistance exercise induced a similar VE•VCO_2_^−1^ slope and OUES compared with endurance exercise at the same metabolic intensity (lactate threshold intensity). Rests between sets during a resistance exercise test was a key factor to detecting similar ventilatory efficiency in a resistance exercise and an endurance exercise at a moderate intensity [23]. In other study, we also showed that acute BJ intake improved performance when a rest between exercises was established during a high intensity resistance exercise test in severe anaerobic metabolism [34].

In this study, there was a significant trend to decrease the VE•VCO_2_^−1^ slope after BJ intake compared with the PL (*p* = 0.06) during both routines although the VE•VCO_2_^−1^ slope only declined significantly with a rest between exercises coinciding with improvements in FS performance (first routine). Again, we suspect that the effect of increased plasma NOx levels during the rest between exercises was crucial for improving the VE•VCO_2_^−1^ slope and, consequently, the FS performance during the first routine. During the resistance exercise in severe anaerobic conditions (second routine without a rest), increased circulating plasma NOx levels did not alter ventilatory efficiency and performance.

NO is a powerful vasodilator [41,42] that enhances blood flow under low levels of oxygen and acidic environments [43]. Blood lactate concentrations in both experimental conditions (~18 mmol•L^−1^) confirmed an acidic environment under severe anaerobic conditions with low oxygen availability. High intensity exercise induces the formation of lactate, VCO_2_ and hydrogen ions [H^+^] causing intracellular acidosis [44]. The VCO_2_ is a consequence of augmented mitochondrial production. The accumulation of lactate and [H^+^] is mainly due to intracellular glycolysis [45,46] and both are liberated into extracellular fluid [47,48]. Severe metabolic acidosis induced by high intensity exercises can preserve increased muscle dysfunction by inhibiting mitochondrial activity. In response to increased acidosis, physiological mechanisms to clear lactic acid are based on an increased ventilatory rate to clear accumulated VCO_2_ [49,50].

In this anaerobic environment, it was expected that there would be an elevated VE•VCO_2_^−1^ slope in response to adequate VE and poor perfusion (VE/perfusion mismatching). Compared with other studies, the mean values of the VE•VCO_2_^−1^ slope (BJ, 31.9; PL, 33.8) and the OUES (BJ, 5.7; PL, 5.6) were worsened in our athletes than in highly trained athletes (VE•VCO_2_^−1^ slope, ~28; OUES, 3.8) [2] and than in healthy people (VE•VCO_2_^−1^ slope ~19 to 30; OUES, ~2.6) [8,51,52]. The values of the VE•VCO_2_^−1^ slope exceeding 34 have been used as a reference to determine ventilatory impairment in diseases [53,54] or to indicate an inefficiency of the respiratory system [51]. The VE•VCO_2_^−1^ slope was worsened in the FS exercise during the first routine (BJ = 36, PL = 43.3) and the second routine (BJ, 43.7; PL, 43.9) as the anaerobic environment was raised (Figure 3). The obvious difference in the VE•VCO_2_^−1^ slope between the two experimental conditions was produced in the FS exercise during the first routine and in the subsequent recovery time. Theoretically, VE/perfusion mismatching was aggravated in the PL group during the FS exercise (first routine), coinciding with a decrease in weightlifting performance.

A rational explanation for the coincident improvements in the VE•VCO_2_^−1^ slope and exercise performance during the first routine (with a rest) after BJ intake could be linked to the biochemical and physiological properties of NO. An elevated VE•VCO_2_^−1^ slope was verified in both experimental groups (BJ and the PL); therefore, the mismatching of perfusion/VE was increased during the high intensity resistance exercise in both experimental groups [55]. This physiological event would probably worsen the pulmonary gas exchange by reducing the compliance of the alveoli and by compressing the small blood vessels. As a result, uneven air and blood flow distribution would be expected in the lungs [56]. A 3 min rest between the WBS and FS exercises could have induced a faster NO_3_^−^–NO_2_^−^ conversion to NO after BJ intake. We suggest that NO could have contributed to an increased blood flow and pulmonary vasodilation reversing a possible vasoconstriction [57,58] especially during a rest. Consequently, the efficiency of the pulmonary gas exchange could be improved by decreasing VE for a given CO_2_ output and arterial PCO_2_ [23] after increased plasma NOx concentrations. Therefore, improvements in resistance exercise performance after BJ intake could be related to the matching of VE/perfusion when a rest period is applied between exercises.

The effect of dietary NO_3_^−^ intake on the VE•VCO_2_^−1^ slope and the exercise performance remains controversial. Several studies have proposed that ventilatory efficiency was unchanged in cardiorespiratory diseases after acute dietary NO_3_^−^ intake; however, exercise performance was improved in patients with heart failure but not in patients with chronic obstructive pulmonary disease [59,60]. Exogenous NO precursors have been confirmed to reduce the VE•VCO_2_^−1^ slope in patients with systolic heart failure [26]. Unfortunately, our results cannot be discussed in relation with studies in well-trained athletes because the VE•VCO_2_^−1^ slope was not evaluated after BJ ingestion. Given the limited scientific evidence, the possible effect of the increased circulating plasma NOx levels on the VE•VCO_2_^−1^ slope during endurance and resistance exercises in well-trained athletes warrants further investigation.

It is possible that the perceived differences between studies were due to the proposed protocol, the type of exercise and the checkpoints established during the data analysis. It was evident that the aerobic-anaerobic conditions and the established rest elicited a varied response of the VE•VCO_2_^−1^ slope throughout the test. BJ intake produced an ergogenic effect at specific times depending on the anaerobic conditions of the resistance exercise.

A novel finding observed in this study was that the VE•VCO_2_^−1^ slope was reduced to normal values (BJ and PL < 25) during a rest between exercises and routines in the two experimental conditions; instead, the OUES remained elevated in the two experimental conditions (BJ and PL > 5.2). Surprisingly, the VE•VCO_2_^−1^ slope decreased during the rest between both routines after BJ intake. The reduced VE•VCO_2_^−1^ slope was maintained after the improvements in the FS exercise. It appeared that increased plasma NOx levels caused an ergogenic effect on the VE•VCO_2_^−1^ slope during a rest. This strengthened the theory of the possible vasodilator effect of BJ to regulate VE to remove CO_2_, improving the matching of VE/perfusion during a rest.

Finally, the PetCO_2_ was evaluated to corroborate the ergogenic effect of BJ on the VE/perfusion relationship. It has been demonstrated that the PetCO_2_ decreased during a high intensity exercise coinciding with a reduced blood flow and blood velocity [61,62]. Furthermore, the impairment of the pulmonary blood flow and cardiac output during exercise and augmented physiological dead space were the key causes for reducing the PetCO_2_ in cardiac diseases [18,63].

Oral nitrate supplementation has shown no effect on the PetCO_2_ and time trial performance during normoxia and hypoxia conditions in trained male cyclists [64]. Other studies have yielded contradictory results. Lewis et al. verified no significant effect of an exogenous NO precursor on the PetCO_2_ and the proportion of physiological dead space to tidal volume in patients with systolic heart failure [26]; however, others have found improvements in ventilatory efficiency and the PetCO_2_ in patients with pulmonary hypertension [20]. There are no studies assessing the behavior of the PetCO_2_ during high intensity resistance exercises after dietary NO_3_^−^ intake. We speculated that BJ improved pulmonary blood flow and cardiac output resulting in better perfusion of the alveoli and increased PetCO_2_ [20].

This study presents a few limitations that should be considered. Minimal changes in performance and physiological responses are usually observed in well-trained athletes [33]; therefore, the sample size should be increased in future research.

## 5. Conclusions

Acute BJ intake improved the VE•VCO_2_^−1^ slope and the PetCO_2_ coinciding with an enhanced resistance exercise performance in well-trained athletes. The positive effect of increased plasma NOx levels on the VE•VCO_2_^−1^ slope and PetCO_2_ could be induced, at least in part, when the aerobic conditions were gradually recovered during a rest. It is likely, therefore, that a rest after an acute resistance performance plays a key role in improving NO_3_^−^–NO_2_^−^ conversion to NO during severe anaerobic exercise with low oxygen availability, enhancing its vasodilator effect at a pulmonary level.

The findings reported here open new lines of research for understanding the physiological mechanisms that link the effects of NO with lung function and exercise performance during high intensity resistance exercises, especially during a rest.

## Data Availability

Data are presented in the manuscript; further information available upon request.
